# Mechanism of Yiqi Huoxue Granule in promoting angiogenesis of skin lesion tissue by increasing CSF2 to reduce mesenchymal stem cell apoptosis

**DOI:** 10.3389/fphar.2026.1649017

**Published:** 2026-01-21

**Authors:** Weili Shi, Shuhui Wang, Shanshan Liu, Zhen Lei, Peishuo Yan, Xinzhou Wang, Chaoqun Lu, Nan Qin, Pengfei Lu

**Affiliations:** 1 The Second Clinical Medical College of Henan University of Chinese Medicine, Zhengzhou, Henan, China; 2 Central Laboratory of Henan Province Hospital of TCM, Zhengzhou, Henan, China; 3 The Third Affiliated Hospital of Henan University of Chinese Medicine, Zhengzhou, Henan, China

**Keywords:** angiogenesis, Csf2, mesenchymal stem cell, skin lesion, Yiqi Huoxue Granule

## Abstract

**Objective:**

To explore the effect and mechanism of the Yiqi Huoxue Granule in improving the survival of mesenchymal stem cells (MSCs) induced by hypoxia and promoting angiogenesis in damaged tissues.

**Methods:**

Animal experiments: A rat skin injury model was established. The skin healing degrees were compared among the model group, MSC group, and Yiqi Huoxue Granule group and Yiqi Huoxue Granule combined with MSC group. The intensity of DIR-labeled MSCs was observed by frozen section, and the expression of CD31 in tissues was detected by immunofluorescence. Cell experiments: A hypoxic MSC model was constructed. The effect of the Yiqi Huoxue Granule on the viability of hypoxic MSCs was detected by CCK8. Flow cytometry was used to observe the apoptosis rate of MSCs. The effect of Yiqi Huoxue Granule on tube formation of MSCs was observed by tube formation assay. Differentially expressed genes were analyzed using transcriptomics and verified through RT-PCR. Key factors were analyzed by PPI.

**Results:**

DIR-labeled MSCs showed that at a concentration of 50 μM, the count of red spots in MSCs significantly increased compared to 12.5 μM and 25 μM (P < 0.01 or 0.001), and there was no statistical difference in cell viability compared to the 12.5 μM and 25 μM groups (P > 0.05), thus 50 μM DIR was selected for *in vivo* tracing. On the 11th day of intervention, compared to the model group, MSC group, and Yiqi Huoxue Granule alone group, the wound diameter in the Yiqi Huoxue Granule combined with MSC group was significantly reduced (P < 0.05). On the 7th day of intervention, the percentage of CD31 fluorescence area in the Yiqi Huoxue Granule combined with MSC group was significantly increased compared to the MSC group (P < 0.01), and compared to the MSC group, the MSC combined with Yiqi Huoxue Granule increased the MSC DIR fluorescence area and intensity (P < 0.05). Cell experiment results showed that compared to the hypoxic model group, high-dose Yiqi Huoxue Granule reduced MSC apoptosis (P < 0.001) and promoted lumen formation. Transcriptomic analysis identified 19 apoptosis-related genes linked to hypoxia. Following RT-PCR and PPI analysis, 9 genes centered around Csf2 were selected. Among these, Csf2, Il1a, Il6, Fgf10, and Cd274 were found to be upregulated, while Ccl2, Pde1a, Nptx1, and Igfbp3 were downregulated.

**Conclusion:**

This research offers a novel perspective for MSC apoptosis using Yiqi Huoxue Granule. Yiqi Huoxue Granule promotes the survival of MSCs under hypoxia and in damaged skin tissue, improves angiogenesis in damaged skin tissue, accelerates skin wound healing, and is closely related to the elevation of Csf2.

## 1 Introduction

Skin injuries are a prevalent clinical issue, with millions of cases reported annually worldwide (Jani et al., 2023). These injuries can lead to significant morbidity, affecting patients’ quality of life and imposing a substantial burden on healthcare systems. The healing process is complex and can be hindered by various factors, including infection, poor blood supply, and underlying health conditions. Current clinical treatments for skin wounds, such as surgical interventions, dressings, and topical agents, often yield inconsistent results and may not adequately promote healing in all patients (Abbasi et al., 2020). Furthermore, the use of stem cell therapies, particularly MSCs, has emerged as a promising avenue for enhancing wound healing due to their regenerative properties. However, challenges remain regarding the viability and efficacy of MSCs in hypoxic environments, which are common in chronic wounds (Alwohoush et al., 2024; Hwang et al., 2020). Traditional Chinese medicines supply feasible method for MSCs transplantation and survival. The research group has long been committed to the basic research of Yiqi Huoxue prescription, which has found that in addition to its anticoagulant and anti-inflammatory effects, it can promote angiogenesis in hypoxic endothelial cells through the miR-126 mediated signaling pathway (Xinzhou et al., 2020; Gao et al., 2021). Traditional Chinese medicines that promote blood circulation and remove stasis can effectively mobilize and promote the homing of stem cells to ischemic myocardial tissue, enhancing the repair capacity of ischemic tissues (Yuan et al., 2024; Shi et al., 2019; Chunmiao et al., 2024). However, there has been no report on the curative effect of Yiqi Huoxue prescription on skin injury. Therefore, this study will use rat models with skin injury and hypoxic MSCs to investigate the role and mechanism of Yiqi Huoxue prescription in reducing stem cell apoptosis and repairing damaged skin, to provide a reference for the application of Yiqi Huoxue Granule.

## 2 Methods

### 2.1 Materials

#### 2.1.1 Experimental animals

20 Male SD rats weighing 180–200 g, specific pathogen-free (SPF) grade, purchased from Sipeifu (Beijing) Biotechnology Co., Ltd. (Certificate No.: SCXK (Jing)-2019-0010). The rats were reared in the Central laboratory of Henan Provincial Hospital of Traditional Chinese Medicine with license number SYXK (Yale) 2021-0018. The laboratory was kept under 12 h light, room temperature (25 ± 2) °C, humidity (45 ± 10)%, and the rats were fed freely. The experimental protocol was approved by the animal ethics board of Henan Provincial Hospital of Traditional Chinese Medicine, Approval No. is PZ-HNSZYY-2024-044.

#### 2.1.2 Experimental medications and reagents

Yiqi Huoxue Granules were produced by Sichuan New Green Pharmaceutical Technology Development Co., Ltd. The daily dose of Yiqi Huoxue granules per person is 8 g, which is equivalent to 70 g of raw herbs. The components of Yiqi Huoxue granules were identified by Liquid Chromatography - Mass Spectrometry/Mass Spectrometry, and the identification results are shown in the annex.

#### 2.1.3 Experimental instruments

CO2 incubator (Thermo Fisher Scientific, USA, 3110 series), microplate reader (Thermo Fisher Scientific, China, FC model); flow cytometer (BD Company, USA, FACS Jazz); fluorescence microscope (Olympus, Japan, CKX41); ultra-micro nucleic acid and protein quantifier (Onedrop, China, OD-2000+); gene amplification instrument (BIO-RAD, USA, XBCX-S-25); cryostat microtome (Thermo Fisher Scientific, USA, Cryostar NX50); Incucyte Zoom (Essen Bioscience, USA, 40733), scanner (3DHISTECH, Hungary, Panoramic MIDI); Gene Amplifier (BIO-RAD, USA, DNA engine XBCX-S-025), Real-time PCR System (ABI, USA, 7500 Fast).

### 2.2 Animal experiments

#### 2.2.1 DIR fluorescent labeling of MSC

When cells confluence reached 90%, they were digested with trypsin, centrifuged, and then resuspended at a density of 1 × 10^6^ cells/mL in 600 μL of complete medium containing different concentrations of DIR dye (KeyGen, China, lot: 20241122). Cells were incubated at 37 °C for 30 min. After centrifugation at 1,500 r, the supernatant was discarded, and cells were collected. After cells were washed twice with PBS, they were cultured in complete medium. Zoom photography was performed at 2, 12, and 24 h respectively. The cells labeled red by DIR were MSCs.

#### 2.2.2 Cell counting Kit-8 assay for cell viability detection

After 24 h of culture, the cell supernatant was discarded, and serum-free medium containing 10% CCK-8 (Meilunbio, China, MA0218-l-Arr-04E) was added. After incubated at 37 °C for 2 h, the optical density (OD) value of the cells was measured at 450 nm using a microplate reader.

#### 2.2.3 Establishment of skin lesion model

After anesthetizing the rats, remove hairs from the back, and prepare a circular full-thickness skin defect wound with a diameter of 2.0 cm on left side of the spine, exposing the wound and maintaining the hygiene of the dressing (Committee, 2018).

#### 2.2.4 Grouping and dosage for gastric administration

20 rats were divided into four groups, with 5 rats in each group. The calculation of the sample size is referenced in the literature (Ce et al., 2023). The experimental groups included the model group, the MSC group, the Yiqi Huoxue Granule group, and the MSC + Yiqi Huoxue Granule group. The conventional dosage of Yiqi Huoxue Granule (0.715 g/kg/d) for rats is converted according to the conversion factor between rat and human body surface area, and twice the conventional dose is used as the actual dosage for this study (1.43 g/kg/d) (Xinzhou et al., 2020). Immediately after modeling, the MSC group and the MSC + Yiqi Huoxue Granule group were subcutaneously injected with 40 μL of 1.5 × 10^6^ MSC suspension labeled with DIR at four sites. The model group were injected with PBS at the same sites. On the next day, the Yiqi Huoxue Granule group and the MSC + Yiqi Huoxue Granule group were gavaged with the above - mentioned dose, and the model group and MSC group were gavaged with an equal volume of physiological saline. The administration lasted for 14 days.

#### 2.2.5 Observation of skin healing

The wound diameter was measured with a ruler on the 3rd, 7th, and 11th days after modeling.

#### 2.2.6 Observation of skin angiogenesis in each group

After the tissue was embedded in paraffin blocks and sectioned, antigen retrieval was performed. Then, a histochemical pen was used to draw a circle around the tissue. The tissue was incubated with a spontaneous fluorescence quencher for 5 min, rinsed with running water, and then incubated with BSA in the circle for 30 min for blocking. The primary antibody against CD31 (Servicebio, China, Gb113151) was added and incubated overnight at 4 °C in a wet chamber. After washing, the secondary antibody was added and incubated at room temperature for 50 min in the dark. DAPI staining solution (Biosharp, China, lot: 22104336) was added to the circle to stain the nuclei and incubated at room temperature for 10 min in the dark. After gentle washing twice and slightly drying by shaking, the sections were mounted with an anti - fluorescence quenching mounting medium. Fluorescence scanning was performed using a scanner, and the average optical density of CD31 was analyzed using ImageJ.

#### 2.2.7 Survival of MSCs in each group

The skin lesion samples were frozen into ice blocks in liquid nitrogen and then sectioned using a cryostat microtome. The sections were left at room temperature for 30 min, fixed in acetone at 4 °C for 10 min, dried in an oven for 10 min, washed three times with PBS, and then subjected to antigen retrieval. After natural cooling at room temperature, DAPI working solution was added to stain the nuclei for 10 min at room temperature. After mounting, the sections were scanned, and the fluorescence intensity of DIR was analyzed using ImageJ.

### 2.3 Cell experiments

#### 2.3.1 *In vitro* culture method and identification of bone marrow MSCs

Bone marrow MSCs of rat pups were extracted using the whole - bone marrow adherent method. Adipogenic and osteogenic kits were used for induction and identification, and flow cytometry was employed to identify stem cell surface markers in our previous work (Weili et al., 2024).

#### 2.3.2 Construction of hypoxia model

A hypoxia model was established using the AnaeroPack reagent bag (Mitsubishi, Japan, lot: 4177LH - 2) and a hypoxia - sealed chamber from Mitsubishi Gas Chemical (MGC). Immediately placed the opened anaerobic reagent bag into the culture tank and cover it with the sealing lid, because the reagent bag would absorb oxygen and produce carbon dioxide immediately upon contact with air. Within 1 h, the oxygen concentration decreased to below 0.1%, and a hypoxic state with a carbon dioxide concentration of approximately 5% was achieved.

#### 2.3.3 Grouping of cell experiments

The Yiqi Huoxue Granule were ultrasonically dissolved, filtered through a 200 - mesh filter sieve, and then stored in freezer. Cells were divided into the normal group, hypoxia model group, VEGF group, low - dose Yiqi Huoxue Granule group (l-YQHX), medium - dose Yiqi Huoxue Granule group (m- YQHX), and high - dose Yiqi Huoxue Granule group (h- YQHX). Among them, the cell administration doses of low-dose, medium-dose and high-dose Qi Yi Huo Xue Granule were 0.25 mg/mL, 0.5 mg/mL, and 1 mg/mL respectively (Gao et al., 2021). The normal group and the model group were intervened with low-glucose medium containing 2% fetal bovine serum. The VEGF group was intervened with VEGF (Peprotech, USA, 1107436 L0612) at a concentration of 20 μg/L prepared. The low - dose, medium - dose, and high - dose Yiqi Huoxue Granule groups were treated with the Yiqi Huoxue Granule at concentrations of 0.25 mg/mL, 0.5 mg/mL, and mg/mL, respectively.

#### 2.3.4 CCK8 assay

Inoculate 5,000 cells in 100 μL into each well of a 96 - well plate for culture. When the cell confluence reaches 70%–80%, divide the cells into groups for intervention. After 24 h, discard the culture medium. Add 100 μL of serum-free medium containing 10% CCK8 (meilunbio, MA0218 - L - Apr - 04E) to each well and incubate in the incubator for 2 h. Measure the absorbance of each well at a wavelength of 450 nm using a microplate reader. The cell survival rate (%) = [(Absorbance of experimental group A - Absorbance of blank well)/(Absorbance of experimental group B - Absorbance of blank well)] × 100% (Group A includes the hypoxia model group and the drug - intervention group, while group B is the normal control group).

#### 2.3.5 Flow cytometry analysis

Seed the cells into a 6 - well plate (2 mL per well) at a cell concentration of 5 × 10^4^/mL. When the cell confluence reaches 70%–80%, divide the cells into groups for intervention as described above. After 24 h of hypoxia intervention, use the Annexin V - FITC/PI double - staining cell apoptosis detection kit (KeyGen Biotech, China, lot: 20211224) for detection. The steps are as follows: Aspirate the supernatant, wash the cells with PBS, then add 500 μL of EDTA - free trypsin (Solarbio, China, lot: 240008008) to each well for digestion for 10 min. Gently pipette the cells 5 times, add 1 mL of PBS, and centrifuge the mixture (including the supernatant) at 2000 r/min for 5 min. Wash the cells twice with PBS and centrifuge, then discard the supernatant. Add 500 μL of Binding Buffer, 5 μL of Annexin V - FITC, and 5 μL of Propidium iodide to each tube in sequence, mix well, and incubate at room temperature in the dark for 10 min. Transfer the samples to a flow cytometer for detection and use FlowJo for statistical analysis.

#### 2.3.6 Tube formation assay

Place Matrigel matrix gel (Corning, USA, 356,231), culture plates, and 200 - μL pipette tips at 4 °C overnight. The next day, add the matrix gel to a 96 - well plate at 50 μL per well on ice and place it in the incubator for 40 min until it solidifies. Resuspend the digested cells and adjust the cell density to 1 × 10^5^/mL. Add 200 μL of the cell suspension to each well. After 1 h of culture for cell attachment, divide the cells into groups for intervention. After 10 h, randomly select 5 fields of view under an inverted microscope for photography, and analyze the tube mesh using ImageJ.

#### 2.3.7 Detection of hypoxia - inducible factor 1 alpha (Hif1α) and L-lactate dehydrogenase (L-LDH) in cell supernatant

Collect the supernatants from the normal group, the model group, and the high-dose Yiqi Huoxue Granule group. Use the Hif1α ELISA kits (Elabscience, China, lot: WZ0548BT9329) and L-LDH (Beyotime, China, lot: A161241128) reagent kit to detect the levels in each group.

#### 2.3.8 Transcriptomic sequencing

The transcriptomic sequencing was completed by Sinotech Genomics Company. In this study, genes with a log_2_ fold change (log_2_FC) greater than 1.5 were considered differentially expressed genes. The raw database can be downloaded from the NCBI official website (Accession number PRJNA1277166).

#### 2.3.9 RT-PCR

Total RNA was extracted using the column - based method (Sangon Biotech, China, B518651). RNA reverse transcription was performed using the PrimeScriptTM RT Reagent Kit (Perfect Real time) (TakaRa, Japan, lot AKF1580A): 1. Reaction system: 7 μL of template RNA, 1 μL of 5X PrimeScript Buffer (for Real Time), 2 μL of PrimeScript RT Enzyme Mix I, 1 μL of Oligo dT Primer, 4 μL of Random 6 mers, 1 μL of RNase Free dH_2_O, and 4 μL of EASY Dilution (for Real Time PCR). Gently mix the reagents, centrifuge briefly, and then place the reaction on gene amplifier. 2. Program: Incubate at 37 °C for 15 min, 85 °C for 5 s, and then take out the reaction at 4 °C. Real - time PCR was carried out using TB Green® Premix Ex Taq™ II (Tli RNaseH Plus) (TakaRa, Japan, Lot AL61804A): 1. Reaction system: 7.5 μL of TB Green Premix Ex Taq I (Tli RNaseH Plus), 0.6 μL of Forward Primer, 0.6 μL of Reverse Primer, 0.3 μL of ROX Reference Dye, 4.5 μL of DDH_2_O, and 1.5 μL of the reverse - transcribed sample. 2. Program: Pre - denaturation at 95 °C for 30 s; amplification (40 cycles) at 95 °C for 5 s and 60 °C for 34 s. The results were calculated and analyzed according to the 2^−ΔΔCt^ method. All gene primers were designed and synthesized by Sangon Biotech. The gene primer sequences are shown in Table 1.

**TABLE 1:** Gene primer sequences.

Gene name	Sequence (5′-3′)	Sequence (5′-3′)
FGF10	F:GGGGAAACTCTATGGCTCAAAAG	R:AGGGGAAACTCTATGGCTCAAAAG
Il1a	F:CGCTTGAGTCGGCAAAGAAAT	R:GCTTGAGTCGGCAAAGAAATCA
Ccl2	F:TGTCTCAGCCAGATGCAGTT	R:AGCCAACTCTCACTGAAGCC
Il6	F:GCCCACCAGGAACGAAAGTC	R:ACAAGTCCGGAGAGGAGACT
Csf2	F:TGGCGCCTTGACCATGATAG	R:GACCCGCCTGAAGCTATACA
Cdh13	F:GCCGGTCCTAAACTTGACCT	R:AGCCGGTCCTAAACTTGACC
Htr2a	F:GCAATTAGGTGATGGCCCGA	R:AATTAGGTGATGGCCCGAGG
RT1-CE16	F:AGTAGGAGTCTACGCCCCTG	R:TAGGAGTCTACGCCCCTGC
Pcsk5	F:TGGCCCAGGATTCAAGAACT	R:CTGCAAGGATGCAACGGAA
Pde1a	F:GCCTGAACAGTCGGTCAGTA	R:GGATAGCCTGAACAGTCGGT
Cd274	F:TCCTCGCCTACAGGTAAGTCT	R:CTCCTCGCCTACAGGTAAGTC
Nptx1	F:ACTGGAGAACCTCGAGCAGT	R:TGGCTCGGGCAAGAATACG
Igfbp3	F:AGCCAGCGCTACAAAGTTGA	R:ACAGCCAGCGCTACAAAGTT

#### 2.3.10 PPI analysis

The Protein - Protein Interaction (PPI) analysis of the target genes in the transcriptome was performed using the STRING database (https://string-db.org/).

### 2.4 Statistical analysis

The data were analyzed using SPSS 21.0. All data were presented as the mean ± standard deviation (x̅ ± s). We used one-way analysis of variance (ANOVA) for multiple independent samples with homogeneous variances, and the rank-sum test for those with heterogeneous variances. For two independent samples, we used the T-test for homogeneous variances and the Welch’s T-test for heterogeneous variances. P - value less than 0.05 was considered statistically significant.

## 3 Experimental results

### 3.1 Animal experiments

#### 3.1.1 DIR fluorescent concentration

**FIGURE 1:**
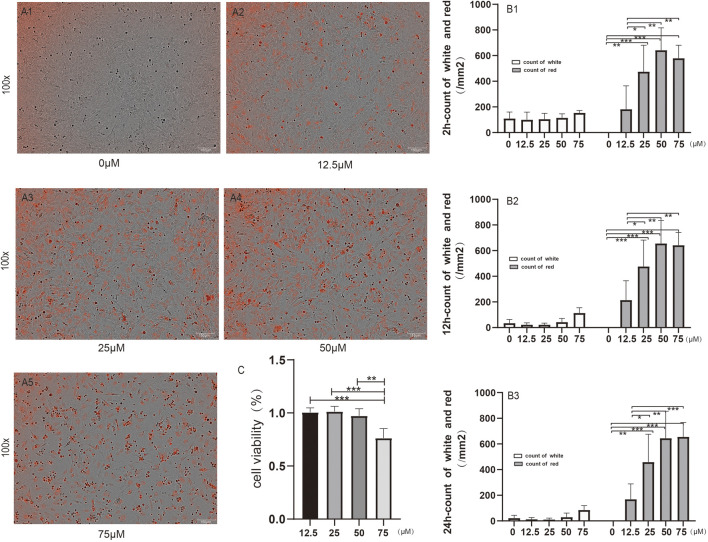
Concentration screening of DIR. Note: **(A1–5)** show the morphology of MSCs under the microscope at 24 h after plating with different concentrations of DIR; **(B1–3)** show the white - light and red - light counts at 2, 12, and 24 h after plating respectively; **(C)** shows the cell viability detected by CCK - 8 assay at 24 h after plating; n = 4; ^*^P < 0.05, ^**^P < 0.01, ^***^P < 0.001.

DIR is a commonly used in - vivo tracing dye. The staining efficiency and the impact on cell viability were observed at 2, 12, and 24 h after plating cells stained with DIR at concentrations of 12.5, 25, 50, and 75 μM respectively (Figures 1A1–5). It was found that compared with 12.5 and 25 μM DIR concentrations, the count of red points and the percentage of red fluorescence of MSCs were significantly increased at 50 μM (P < 0.01 or 0.001). At 12 h and 24 h, there was no significant difference in the red count and percentage between 75 μM and 50 μM (P > 0.05) (Figures 1B1–3), which might be due to the unwashed floating dead cells. After discarding the supernatant, cell viability was detected by CCK - 8 assay. The results showed that the cell viability in the 75 μM DIR group was significantly lower than that in the 12.5, 25, and 50 μM DIR groups (P < 0.01 or 0.001), while there was no statistical difference in cell viability between the 50 μM DIR group and the 12.5, 25 μM groups (P > 0.05) (Figure 1C). Therefore, 50 μM DIR was selected for subsequent in - vivo tracing.

#### 3.1.2 Observation of skin healing

**FIGURE 2:**
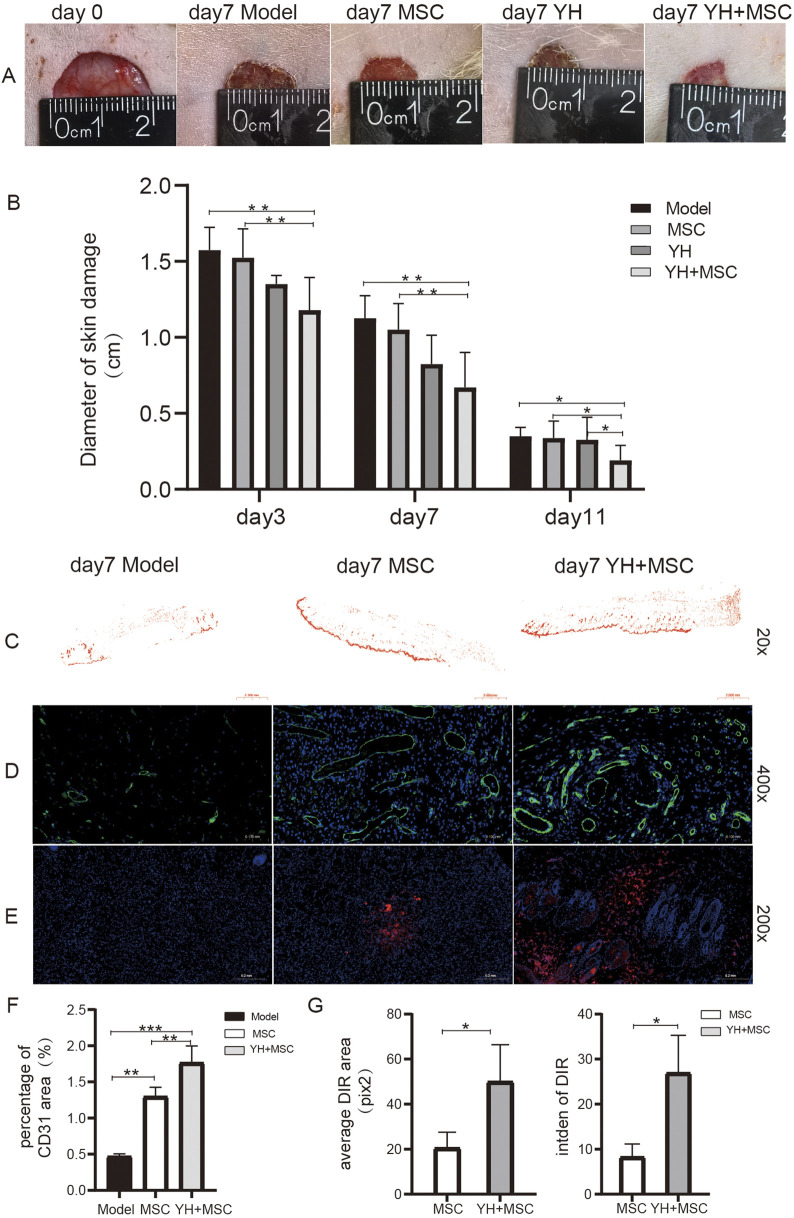
Yiqi Huoxue promotes the survival of MSC and angiogenesis in the lesion tissues. Note: **(A)** Wound healing diagram of lesional tissue; **(B)** Data analysis of wound healing of lesional tissue; n = 4; **(C)** Fluorescent panoramic view of CD31 after ImageJ conversion; **(D)** CD31-labeled blood vessels with green fluorescence. **(E)** DIR fluorescence intensity of frozen sections; **(F)** Percentage of CD31 fluorescence area, n = 3; **(G)** Intden (intensity and density) of DIR fluorescence, n = 3; ^**^P < 0.01, ^***^P < 0.001.

The wound healing was observed on the 3rd, 7th, and 11th days. It was found that the wound - healing diameter decreased in all groups over time. However, compared with the model group, the wounds in the MSC group, the Yiqi Huoxue prescription group, and the Yiqi Huoxue prescription combined with MSC group healed faster. Specifically, on the 3rd and 7th days of intervention, the wound diameter in the Yiqi Huoxue prescription combined with MSC group was significantly smaller than that in the model group and the MSC group (P < 0.01). On the 11th day, compared with the model group, the MSC group, and the Yiqi Huoxue prescription group, the wound diameter in the Yiqi Huoxue prescription combined with MSC group was significantly smaller (P < 0.05) (Figures 2A,B).

#### 3.1.3 Angiogenesis in the skin

The effect of the Yiqi Huoxue prescription combined with MSCs on CD31 in skin lesion tissues was compared. The results indicated that on the 7th day of intervention, compared with the model group, the percentage of CD31 fluorescent area in the MSC group and the Yiqi Huoxue prescription combined with MSC group was significantly increased (P < 0.01). Compared with the MSC group, the CD31 percentage in the Yiqi Huoxue prescription combined with MSC group was significantly increased (P < 0.01) (Figures 2C,D,F), suggesting that the Yiqi Huoxue prescription combined with MSCs can synergistically promote wound angiogenesis.

#### 3.1.4 Survival of MSCs in the skin

The fluorescence area and intden of transplanted MSCs was observed by frozen - section. It was found that compared with the MSC group, both the area and intden of DIR fluorescence in the Yiqi Huoxue prescription combined with MSC group were increased (P < 0.05), suggesting that the Yiqi Huoxue prescription can promote the survival of in - vitro transplanted MSCs (Figures 2E,G).

### 3.2 Cell experiments

#### 3.2.1 Reduction of hypoxia - induced MSC apoptosis

**FIGURE 3:**
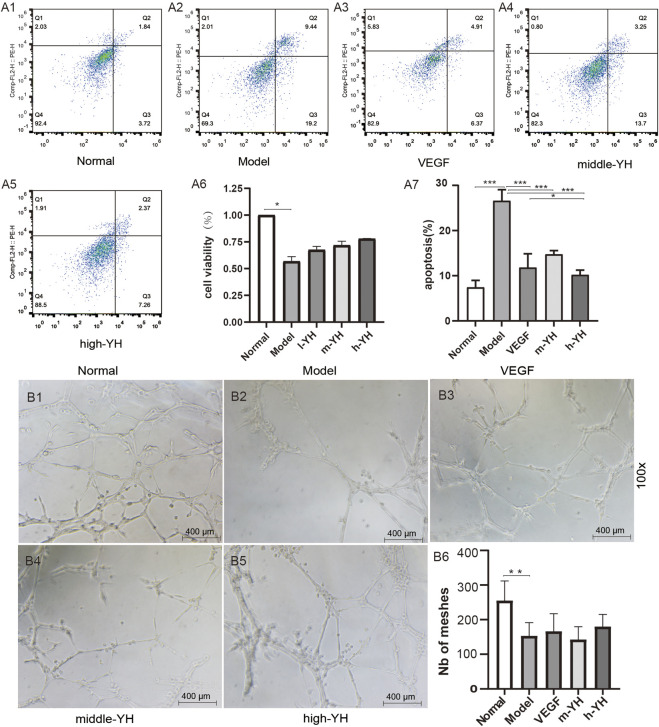
Yiqi Huoxue Granule alleviates hypoxia - induced MSC apoptosis and promotes tube formation. Note: **(A1–5)** Flow cytometry diagrams; **(A6)** CCK8 result analysis diagram, n = 3; **(A7)** Flow cytometry result analysis diagram, n = 3; **(B1–5)** Three - dimensional tube formation diagrams; **(B6)** Tube formation result analysis diagram, n = 5; ^*^P < 0.05, ^**^P < 0.01, ^***^P < 0.001.

The CCK8 assay was used to detect the effect of different doses of Yiqi Huoxue Granule on cell viability. It was found that the viability of MSCs in the model group was significantly lower than that in the normal group (P < 0.05). After the intervention of low, medium, and high doses of Yiqi Huoxue Granule, the cell viability increased, although there was no statistical difference (P > 0.05) (Figure 3A6).

The Annexin V - FITC/PI kit was used to detect the apoptosis of hypoxic MSCs. The results showed that compared with the normal group, the apoptosis rate of cells in the model group increased significantly (P < 0.001). Compared with the model group, the apoptosis rates in the VEGF group, the medium - and high - dose groups of Yiqi Huoxue Granule decreased (P < 0.001) (Figures 3A1–5,A7).

#### 3.2.2 Promotion of tube formation

After coating Matrigel in a 96 - well plate, the effect of Yiqi Huoxue Granule on the three - dimensional tube formation of hypoxic MSCs *in vitro* was observed. It was found that compared with the normal group, the number of meshes in the model group decreased significantly (P < 0.05). Compared with the model group, the number of lumens in the VEGF group and the high - dose group of Yiqi Huoxue Granule increased, although there was no statistical difference (P > 0.05) (Figures 3B1–6).

#### 3.2.3 HIF-1α and LDH levels in cell supernatant

**FIGURE 4:**
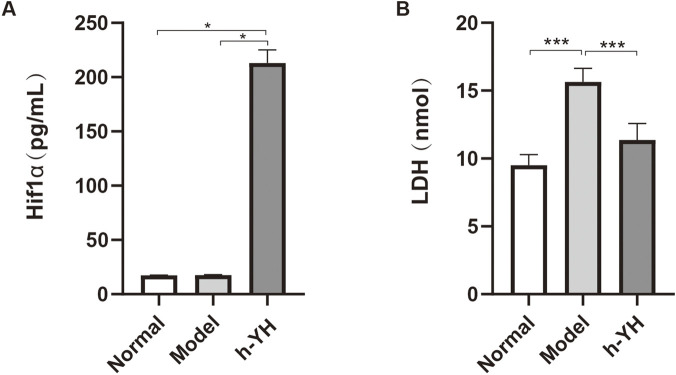
The influence of Yiqi Huoxue Granule on Hif1α and LDH. Note: **(A)** Hifa level in supernatant, n = 3; **(B)** LDH level in supernatant, n = 4; ^*^P < 0.05, ^***^P < 0.001.

After intervention of hypoxic MSCS with the Yiqi Huoxue Granule, the Hif1α in the supernatant of the drug group was significantly higher than that of the normal group and the model group (P < 0.05) (Figure 4A). In contrast, LDH levels in the Model group were markedly higher than those in the Normal group (P < 0.001), while Yiqi Huoxue Granule intervention resulted in a substantial reduction of LDH compared to the Model group (P < 0.001) (Figure 4B).

#### 3.2.4 Transcriptomics

**FIGURE 5:**
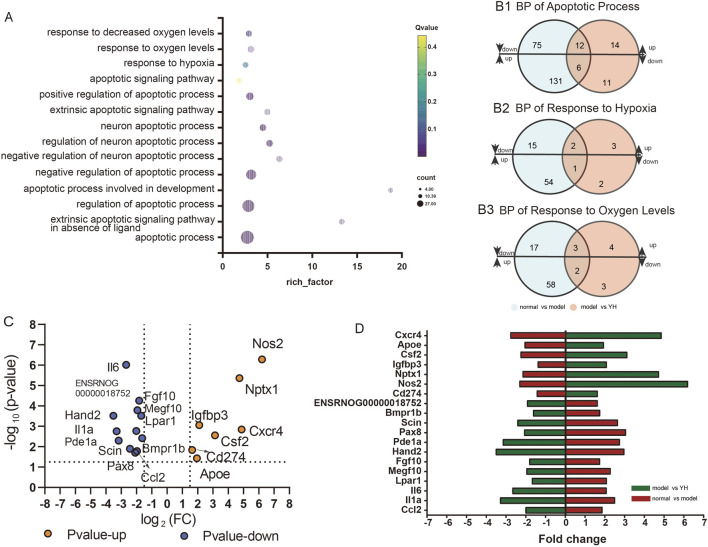
Transcriptomic results of Yiqi Huoxue Granule in the intervention of hypoxic MSC. Note: **(A)** Bubble chart of apoptosis - related biological processes; **(B)** Screening of apoptosis - related genes; **(C)** Volcano plot of apoptosis - related differential genes; **(D)** Bar chart of apoptosis - related differential genes.

The above studies suggested that the high - dose group of Yiqi Huoxue Granule had the best effect on reducing hypoxia - induced MSC apoptosis. Therefore, the MSCs intervened with the high - dose Yiqi Huoxue Granule were used for transcriptomic detection. This study focused on the effect of Yiqi Huoxue Granule on the apoptosis of hypoxic MSCs. Therefore, the pathways related to hypoxia and apoptosis, including “apoptotic process”, “response to hypoxia”, and “response to oxygen levels”, were selected. A bubble chart was made for the top 14 differential biological processes, as shown in Figure 5A. The differential genes related to “apoptotic process”, “response to hypoxia”, and “response to oxygen levels” were analyzed respectively. The analysis methods were as follows: 1. The differential genes in the above three biological processes were screened out for the normal control group vs. the model group and the model group vs. the Yiqi Huoxue Granule group respectively. 2. The intersection of the two groups of genes with opposite expression trends was taken. 3. The duplicate genes were removed. A total of 7 upregulated genes and 12 downregulated genes were obtained (Figures 5B1–3). A volcano plot and a bar chart were used to display the 19 differentially expressed genes respectively, as shown in Figures 5C,D.

#### 3.2.5 PCR verification and PPI analysis

**FIGURE 6:**
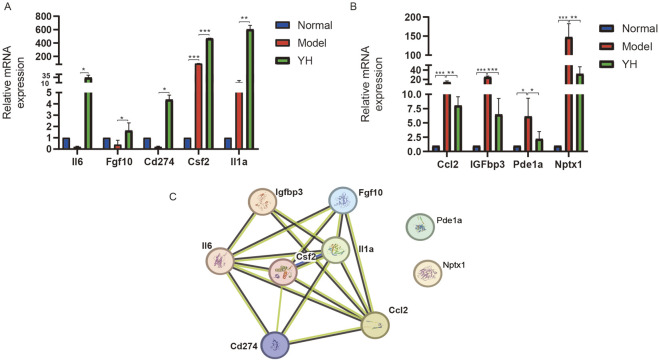
Results of PCR verification and PPI analysis. Note: **(A)** Genes with increased relative mRNA expression, n = 3; **(B)** Genes with decreased relative mRNA expression, n = 3; **(C)** PPI analysis of target genes. ^*^P < 0.05, ^**^P < 0.01, ^***^P < 0.001.

RT - PCR was used to verify the above genes. It was found that 5 genes, namely, interleukin-6 (Il6), colony-stimulating factor 2 (Csf2), fibroblast growth factor 10 (Fgf10), cluster of differentiation 274 (Cd274, is also known as PD - L1), and interleukin-1 alpha (Il1a), were up–regulated after Yiqi Huoxue Granule interventioned, and 4 genes, namely, chemokine (C-C Motif) ligand 2 (Ccl2), phosphodiesterase 1A (Pde1a), neuronal pentraxin 1 (Nptx1), and insulin-like growth factor binding protein 3 (Igfbp3), were downregulated. With the help of PPI analysis, it was found that except for Pde1a and Nptx1, the other factors formed a network diagram centered on Csf2, suggesting that Csf2 is closely related to the effect of Yiqi Huoxue Granule on reducing MSCs under hypoxic conditions (Figure 6).

## 4 Discussion

The Yiqi Huoxue Granule consists of Astragalus membranaceus, Ginseng, Radix Paeoniae Rubra, and Saffron. In the formula, Ginseng and Astragalus membranaceus tonify qi; Radix Paeoniae Rubra, with a bitter taste and slightly cold nature, has the effects of clearing heat, cooling blood, dissipating blood stasis, and relieving pain; Saffron, with a neutral nature and sweet taste, can promote blood circulation and dredge collaterals. The combination of these four herbs together achieves the effects of replenishing qi, activating blood circulation, and generating new tissues. Previously, our team has confirmed that this formula has anti-inflammatory and pro-angiogenic effects, but its impact on hypoxic stem cells has not yet been studied (Xinzhou et al., 2020; Gao et al., 2021). In hypoxic models, MSC apoptosis increases over time in a dependent manner. The high rate of apoptosis under hypoxic conditions severely affects the application effects of MSCs in regenerative medicine and disease treatment (Marolt Presen et al., 2019; Chen et al., 2014; Shen et al., 2019), and there is an urgent need to conduct in-depth research to find effective intervention strategies. Therefore, this study uses a rat skin injury model and an hypoxic MSC model to investigate the effects and mechanisms of Yiqi Huoxue decoction in reducing apoptosis of MSCs under hypoxic conditions.

The findings indicate that the Yiqi Huoxue Granule significantly enhances the healing of skin lesions in rats, increases the survival of implanted MSCs, and promotes angiogenesis in the damaged tissue. The transcriptomic analysis revealed a set of apoptosis-related genes influenced by hypoxia, with a particular focus on Csf2 as a central molecule. Csf2 plays an important role in regulating immune responses, inflammatory responses, and the tumor microenvironment, especially under hypoxic conditions, where changes in its expression and function significantly impact disease progression. With the deepening research on CSF2, more and more evidence suggests that its effects on MSCs in hypoxic environments have important clinical significance, providing new ideas and directions for regenerative medicine.

### 4.1 Role of Csf2 under hypoxic conditions

Under hypoxic conditions, the expression of CSF2 usually increases, which may be an adaptive response of cells to the hypoxic environment (Qi et al., 2023). CSF2 is not only a potent factor for mobilizing the migration and homing of MSCs and promoting tissue repair (Kim et al., 2019; Truong et al., 2017; Alsharidah et al., 2024), but also plays an important role in regulating cellular immune responses, inflammatory responses, and the tumor microenvironment (Becher et al., 2016). Therefore, clarifying the mechanism of action of CSF2 under hypoxic conditions and its role in inflammatory and immune responses and tissue repair is crucial for understanding its potential applications in regenerative medicine.

### 4.2 Hypoxia promotes Csf2 secretion and its effects on Il - 1α, Il - 6, and Ccl2

Hypoxia can promote the expression of Csf2 by activating the Hif - 1α (hypoxia - inducible factor - 1α) signaling pathway, thereby affecting the levels of inflammatory factors such as Il - 1α, Il - 6, and Ccl2. Studies have shown that under hypoxic conditions, the upregulation of Csf2 can enhance the expression of Il - 1α and Il - 6, exacerbating the inflammatory response, promoting the infiltration of immune cells, and facilitating cell proliferation and migration (Qi et al., 2023; Saita et al., 2022). In addition, the increase in Ccl2 is also closely related to the upregulation of Csf2. Under hypoxic conditions, Csf2 not only promotes the secretion of Ccl2 but also further aggravates the inflammatory response by enhancing the chemotaxis of monocytes (Saita et al., 2024). Tissue injury and early inflammatory responses are beneficial for tissue angiogenesis (Li et al., 2000). Contrary to the pro - inflammatory effect of Csf2, Li et al. (2020) found that Csf2 alleviates sepsis - induced acute kidney injury by promoting the transformation of macrophages to an alternative phenotype. In this study, it was found that the Yiqi Huoxue prescription increases the levels of Il - 1α and Il - 6 in hypoxic MSCs and decreases the level of Ccl2. The previous research of our group also found that the Yiqi Huoxue prescription can reduce the LPS - induced inflammatory response (Hong et al., 2019), suggesting that the Yiqi Huoxue prescription may regulate inflammatory factors through multiple targets to maintain the homeostasis of the hypoxic environment.

### 4.3 Potential role of CSF2 in hypoxic angiogenesis and its effects on FGF10 and IGFBP3

FGF10 and IGFBP3 play important roles in angiogenesis under hypoxic conditions. FGF10 is an angiogenic factor, and its expression is upregulated in a hypoxic environment (Li et al., 2015). Meanwhile, IGFBP3, as a growth factor - binding protein, can regulate the biological activity of insulin - like growth factor (IGF), affecting cell growth and survival. Under hypoxic conditions, the expression of IGFBP3 also increases, promoting angiogenesis by regulating the IGF signaling pathway (Dallinga et al., 2020). These two factors complement each other in hypoxia - induced angiogenesis. In a hypoxic environment, CSF2 can regulate angiogenesis by affecting the expression of FGF10 and IGFBP3 (L et al., 2021). Specifically, CSF2 can enhance the expression of FGF10, promoting the proliferation and migration of endothelial cells, and simultaneously regulate the level of IGFBP3 to affect the activity of growth factors, further promoting angiogenesis. Therefore, CSF2 affects angiogenesis through multiple mechanisms under hypoxic conditions. Our previous study has demonstrated that the Yiqi Huoxue prescription can promote angiogenesis (Gao et al., 2021). In this study, it was found that while the Yiqi Huoxue prescription increases the level of CSF2, it also increases the level of FGF10 but decreases the level of IGFBP3, suggesting that the Yiqi Huoxue prescription moderately controls the angiogenic factors of hypoxic MSCs.

### 4.4 Interaction between CD274 and CSF2

CD274 (also known as PD - L1) is an important immune checkpoint molecule. Hypoxia is a common feature in the tumor microenvironment and can significantly affect the expression of CD274 and its role in immune escape. Studies have shown that inflammatory factors and transcription factors such as Hif - 1α and Nf - κB in the tumor microenvironment can directly bind to the promoter region of CD274, promoting its transcriptional activity and enhancing the inhibitory effect of tumor cells on the immune system (Cao et al., 2011; Cao et al., 2024; Wang et al., 2023). CSF2 can enhance the expression of CD274 by activating the HIF - 1α signaling pathway (Mi et al., 2021). However, the role of CD274 in the survival or apoptosis of MSCs under hypoxic conditions remains to be studied. In this study, the Yiqi Huoxue prescription increases the expression of CD274, suggesting that the Yiqi Huoxue prescription may play a role in regulating the immune function of MSCs.

The Yiqi Huoxue prescription promotes the repair of damaged skin tissue by regulating the secretion of factors such as CSF2 by MSCs, providing a new perspective for regenerative medicine. Although there are many studies on CSF2, the results of different studies are not always consistent, which poses challenges for us to understand its role in different disease states. Therefore, in future research, more detailed experimental designs and more comprehensive models are needed to explore the interaction and mechanism between CSF2 and inflammatory factors, angiogenic factors, and immune factors in a hypoxic environment. In addition, as the main prescription for treating ischemic diseases, future research should in - depth and optimize the study of the role and mechanism of the Yiqi Huoxue prescription in the repair of tissue damage by MSCs to achieve more effective disease treatment and clinical translation in regenerative medicine.

## Data Availability

The datasets presented in this study can be found in online repositories. The names of the repository/repositories and accession number(s) can be found below: https://www.ncbi.nlm.nih.gov/, Accession PRJNA1277514.
